# Pseudoenhanced Peak Efficiency in Liquid Chromatography–High Resolution Mass Spectrometry Using Multiple Second Derivatives

**DOI:** 10.1021/jasms.5c00315

**Published:** 2026-06-05

**Authors:** Guillaume Laurent Erny

**Affiliations:** a Associate Laboratory i4HB-Institute for Health and Bioeconomy, University Institute of Health Sciences-CESPU, Gandra 4585-116, Portugal; b UCIBIO-Applied Molecular Biosciences Unit, Translational Toxicology Research Laboratory, University Institute of Health Sciences (1H-TOXRUN, IUCS-CESPU), Gandra 4585-116, Portugal

## Abstract

A mathematical approach is introduced to achieve pseudoenhanced peak efficiency in liquid chromatography–high resolution mass spectrometry (LC-HRMS^1^), utilizing second-derivative transformations with respect to time and Gaussian smoothing cycles. The pipeline involved interpolating all MS scans, recorded as profile scans, onto a common *m*/*z* axis, allowing for second-derivative transformations for each *m*/*z* increment with respect to time across the entire data set. Validation using a public study (comprising 1100 compounds analyzed on a Thermo Q Exactive) demonstrates substantial improvements in feature detection and resolution, with qualitative and quantitative benefits. The transformation enables increased peak efficiency by up to 2 orders of magnitude in multicycle implementations without requiring physical hardware enhancements. The approach provides enhanced separation of complex mixtures, reduced background contributions, and increased reliability in untargeted metabolomics and related analyses.

## Graphical abstract

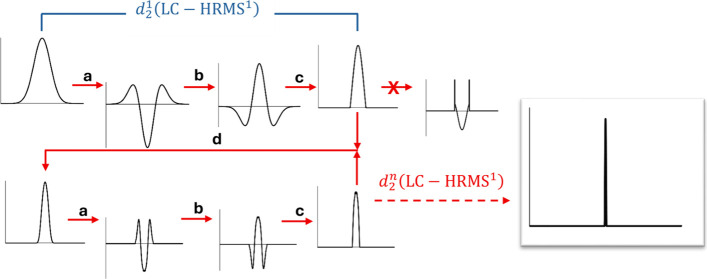

## Introduction

Over the past 20 years, the combination of advanced chromatographic methods, primarily liquid chromatography (LC), gas chromatography (GC), and capillary electrophoresis (CE) with high-resolution mass spectrometry (HRMS) has revolutionized analytical chemistry. This combination enables the sensitive and accurate analysis of complex mixtures in fields such as metabolomics, environmental science, and pharmaceutical research.^1^ A crucial aspect of these methodological advances is the peak efficiency, both chromatographic and mass spectrometric which directly impacts the quality of separation and the specificity, reliability, and accuracy of analytical results.

Enhanced chromatographic peak efficiency, achieved through advances in stationary phase technology, reduced particle sizes, and optimized separation parameters, enabled better and quicker separation of individual analytes, thereby minimizing coelution and ion suppression.^2–4^ Parallel advances in mass spectrometry, including instruments such as the Orbitrap, Fourier-transform ion cyclotron resonance (FT-ICR), and time-of-flight (TOF) analyzers, have significantly improved mass resolving power, allowing for the confident differentiation of isobaric and isomeric species even in highly complex samples.^5,6^ Modern instruments that combine these capabilities, collectively known as LC-HRMS instruments, can separate hundreds of components with concentrations spanning many orders of magnitude in complex matrices within single runs that typically last less than 1 h. These instruments also support various acquisition modes, including full scan mode (also known as LC-HRMS^1^), data-dependent acquisition (DDA), and data-independent acquisition (DIA).^7^

However, these technological benefits bring new challenges. Early chromatographic analyses of hyphenated data primarily employed spectral-based methods, with peak picking performed on two-dimensional spectra that represented the entire three-dimensional data set, such as total ion profiles (TIPs) or total absorbance profiles (TAPs). This method is no longer practical for complex analyses because many features are missed, either hidden near main components, masked by background noise, or obscured by baseline drift. If targeted analysis of expected compounds is relatively straightforward, untargeted analysis, which seeks to detect all elements of a mixture, remains complex and prone to error.

For such complex tasks, analysts rely on computer-assisted tools designed to detect and quantify as many chromatographic-like features as possible.^8,9^ These tools aim to (1) reconstruct two- or three-dimensional features by detecting ions in neighboring scans with similar *m*/*z* values, (2) measure key parameters such as peak centroids, variances, intensities, and signal-to-noise ratios, (3) eliminate features inconsistent with chromatographic-like geometry, and (4) group ions originating from the same compound entering the ionization chamber, including adducts, fragments, and isotopes.^10,11^ Such data processing remains a significant source of error in the analytical workflow, with studies revealing substantial differences between software, which often share fewer than 50% of the identified features.^12–15^

The Finnee MATLAB toolbox was designed for untargeted analysis of LC-HRMS^1^ data sets recorded as profile scans.^16–18^ While MS instruments record scans as continuous traces with many data points across the whole shape of each ion peak (profile scans), most software converts these to centroid scans, which represent peaks as discrete points with single *m*/*z* values and intensities. Although centroiding significantly reduces data size, it can lead to potential information loss and errors.^19–21^ Finnee aims to use the original profile scans but enables faster computation by using linear interpolation to estimate all MS scans across one or multiple experiments onto a common *m*/*z* axis. This method has proven to be reliable and permits complex data transformations such as ensemble averaging.^22^ Although MS spectra are stored independently, Finnee makes it easy to build matrices by aggregating data within small scan ranges. These matrices can then be used to filter and modify the data.^23^

One particularly valuable transformation is the second derivative with respect to elution (separation) time. Such transformations are frequently used in spectral-based analyses for baseline correction. Even derivatives have also been used with chromatographic peaks to enhance resolution. Taking advantage that the *n*th derivative of a Gaussian peak is a polynomial multiplied by the original Gaussian function, Wahab and colleagues,^24^ demonstrated that by combining even derivatives of the same signal (second and fourth derivatives, for example), the resulting peak presents a reduced width while the total area is maintained. This approach was termed resolution-enhanced peak (R.E.P.).

The second-derivative transformation is illustrated in Figure 1, where arrow (a) shows the transformation of a classical Gaussian peak shape into its second derivative. As shown, the original single peak splits into three smaller peaks, the first and last positive and the middle negative. In this work, the aim was to keep the middle peak; this was achieved by multiplying intensity values by minus one (arrow b) and subsequently removing all negative values (arrow c), yielding what was termed pseudoenhanced peak efficiency, an algorithmic improvement in effective peak quality without any physical enhancement by the instrumentation. This does not require peak picking; it is a systematic transformation that applies to the entire trace, regardless of whether one or more peaks are present. As shown in Figure 1, the derivative step cannot be repeated due to the sharp edges of the enhanced peak. However, the application of a peak broadening filter (arrow d) should enable repetition of this process and allow further thinning of the peak. The derivative data set after transformation is denoted as *d*_2_^*n*^(LC-HRMS^1^), where *n* is the number of cycles applied.

**Figure 1:**
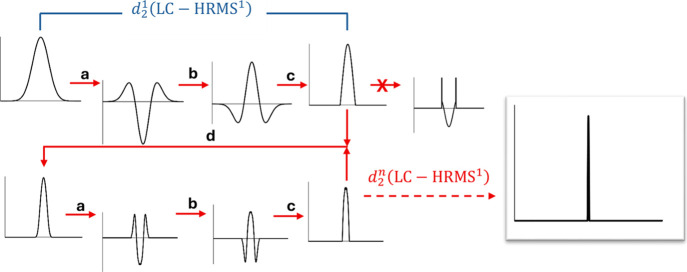
Schematic representation of the proposed transformation leading to derivative data sets. The arrows indicate mathematical steps: (a) second derivative with respect to time, (b) inversion of data, (c) removal of negative values, and (d) application of a peak broadening Gaussian filter.

This article outlines the necessary steps to achieve this transformation with complex data sets acquired by liquid chromatography hyphenated to high-resolution mass spectrometry in full-scan mode (LC-HRMS^1^) with MS scans recorded as profiles. It examines the benefits, limitations, and potential risks of such transformation. To validate the method presented in this manuscript, we used a publicly available benchmark data set developed by Li and colleagues.^13^ This data set includes two well-defined standard mixtures (SA and SB), each analyzed five times, containing a total of 1100 compounds, including drugs and metabolites. Of these, 970 compounds are of equal concentrations in both mixtures, while 130 compounds are divided into six groups with concentration ratios between SA and SB ranging from 1:16 to 16:1. The data set was obtained using two high-resolution mass spectrometry platforms: AB SCIEX TripleTOF 6600 and Thermo Q Exactive HF. For this study, only data from the Thermo Q Exactive mass analyzer were used. This well-characterized benchmark provides an ideal test for evaluating the performance of Finnee and the pseudoenhanced peak efficiency transformation in untargeted LC-HRMS^1^ data analysis, with a focus on feature detection reliability and quantification accuracy.

## Materials and Methods

### Description of Experimental Files and Data Sets

#### Benchmark Data Set 1 (Bd1)

Original LC-HRMS^1^ files from the separation of test compounds using a Thermo Q Exactive HF coupled with a Dionex UltiMate 3000 HPLC system were obtained from the Supporting Information of the work by Li and colleagues.^13^ These raw files (SA*x*.raw and SB*x*.raw, with *x* = 1.5.) were converted to mzML format as detailed below and subsequently processed with the Finnee toolbox. Although the mixture contained 1100 compounds, 836 were detected by Li and colleagues in the final data when using a targeted data analysis approach. The list of detected compounds, including monoisotopic mass, molecular formula, retention time, and fold changes (SB:SA ratio), is available in the Supporting Information of the original article (see Table S8 in Li et al.). The isotopologues of the protonated molecular ions of the 836 compounds were calculated using ChemCalc.^25^ The *m*/*z* values for the [M] + H^+^ and [M + 1] + H^+^ ions and relative intensities of the [M + 1] + H^+^ species relative to the [M] + H^+^ ions are summarized in Table S1 of this manuscript. The separation is characterized by peaks with high noise levels and a very high sampling rate (on average, more than 100 scans per peak). The mass analyzer resolution (*R* = *M*/Δ*M*) was estimated to be approximately 50,000.

#### Exhaled Breath Condensate File (EBC)

This file was used to test the derivative approach with real biological samples. It consists of a pooled quality control sample from a study involving exhaled breath condensates. The chromatographic separation conditions have been described previously.^17^ The sample was analyzed by LC-MS using an Orbitrap Q Exactive Focus (Thermo Scientific) coupled to an Ultimate 3000 UHPLC (Thermo Scientific). The separation is characterized by a few visible peaks in the total ion profile (TIP)[Fn fn1], but good peak shapes and an average scan rate of more than 50 scans per peak. The mass analyzer resolution (*R* = *M*/Δ*M*) was estimated also to be 50,000.

### LC-HRMS^1^ Data Processing

The data processing workflow is outlined as follows. Instrumental raw files are converted to mzML format using msConvert from ProteoWizard,^26^ ensuring that no peak picking filters were used during conversion to preserve profile scan data rather than centroid scans. The mzML files were read and converted using the Finnee toolbox within MATLAB, which generates multiple data sets after specific mathematical transformations (e.g., baseline correction, noise removal, derivatives).^16^ The first data set corresponds to the original, unmodified data. Each MS scan is then linearly interpolated onto a common *m*/*z* and time axis at a constant scan rate. This is done using a two-dimensional linear spline interpolation to maintain a consistent MS data structure and alignment across scans.^22^ This approach preserves the fine structure of Gaussian-shaped peaks, both in the *m*/*z* domain (ion peaks) and the time domain (chromatographic peaks), allowing for efficient data analysis without bucketing or centroid transformation.^16,23^ Unlike the time axis, the extrapolated *m*/*z* axis preserves the variation in sampling density as a function of *m*/*z*, typically proportional to (*m*/*z*)^1.5^ units, which is characteristic of Orbitrap mass spectrometric data.

The derivative data set, the primary focus of this study, was created by dividing the whole data set into multiple zones. Each zone’s data are arranged into two-dimensional matrices with the scan number as the *x*-axis and *m*/*z* values as the *y*-axis. For the test files, the matrices typically contained 500 columns and approximately 550,000 rows. These matrices were processed through iterative cycles of two filters applied over each row: (1) a Gaussian smoothing filter to reduce noise and broaden peaks, and (2) a second derivative filter followed by inversion of intensity values and removal of negative values. The number of cycles and Gaussian filter parameters depend on the original peak width, background noise, and desired final peak width. Gaussian smoothing was chosen for its effective noise reduction and suitability for multiple cycling. While parsing multiple MS spectra into matrices should enable the use of two-dimensional smoothing filters, tests with a two-dimensional Gaussian filter were unsuccessful. Although the computation time increased by more than a factor of 2, the reduction in noise was negligible. The low sampling rate of classical mass analyzers in the *m*/*z* dimension appears to be the limiting factor, as large kernel sizes cannot be used without significantly reducing mass resolution. After filtering, each matrix column was saved as an individual scan in the derivative data set. *d*_2_^*n*^(LC-HRMS^1^), allowing subsequent data analysis methods such as total ion profiles (TIP), base peak profiles (BPP), and extracted ion profiles (XIP). The functions developed for this work are available on GitHub (https://github.com/glerny/Finnee2024) and the Zenodo public repository. A short tutorial and the pseudocode for the derivative function can be found in Supporting Information, S1 and S2 respectively).

## Results and Discussion

### Targeted Analysis with a Single File from the Bd1 Data Sets

The initial validation was performed using the file SA1.raw from the Bd1 data set. Several smoothing filters were tested, but the Gaussian smoothing filter proved to be the most effective. This linear filter applies a Gaussian function as a weighting factor, averaging data points near each point in the signal. The degree of smoothing depends on the Gaussian kernel width: a wider kernel yields stronger smoothing but may slightly broaden peaks. In this study, the Gaussian filter was applied to reduce noise before calculating the second derivative and to smooth the peak edges after derivation. The Gaussian kernel width corresponds to the number of neighboring scans used and should ideally depend on the chromatographic peak width. As described in the Materials and Methods section, all MS scans were interpolated to a common *m*/*z* axis and a new time axis with a constant scan rate. To achieve pseudoenhancement of peak efficiency, the Gaussian kernel width was decreased with each successive cycle. Optimization involved testing different cycle numbers and filter widths through targeted analysis. The highest pseudoenhanced peak efficiency was achieved after 10 cycles, using the following Gaussian kernel widths in scans: 91, 71, 55, 47, 39, 31, 23, 15, 9, and 5, respectively. The initial filter width is approximately equal to the average number of scans per peak. It should be emphasized that, as illustrated in Figure 1, the first step should have been a second derivation. However, MS data are characterized by high noise that should be reduced prior to derivation. In this approach, Gaussian smoothing is used to reduce the sharpening of the peak edge after derivation and data removal, and to control and reduce noise.

Figure 2 shows representative extracted ion profiles (XIP) for selected target compounds. The XIPs were generated by summing all intensities within an *m*/*z* window defined as *m* ± Δ*m*, where *m* is the *m*/*z* value at the peak apex of the protonated molecular ion listed in Table S1, and Δ*m* = 4 × *m*/*R*, with *R* being the mass analyzer resolution (50,000). Inserts A, B, C, and D show compounds C_27_H_31_N_7_OS (compound 4 in Table S1), C_20_H_22_N_8_O_5_ (compound 6 in Table S1), C_23_H_26_O_3_ (compound 220 in Table S1), and C_25_H_23_N_7_OS (compound 626 in Table S1), respectively. For each compound, the first panel displays the XIP from the original data set (black) and after one Gaussian smoothing (red). The second panel with a shifted axis shows the XIP obtained after applying ten derivative cycles (*d*_2_^10^(LC-HRMS^1^) data set). As seen in Figure 2A,B, the *d*_2_^10^(LC-HRMS^1^) data set provides significantly enhanced peak efficiency even with a low signal-to-noise ratio (*S*/*N*). However, overfitting may remain problematic in cases of perfectly isobaric ions that are not baseline-resolved, as shown in Figure 2C, where the *d*_2_^10^(LC-HRMS^1^) XIP displays only one peak although two peaks can be distinguished in the original data set. It should be noted that two peaks are observed in the *d*_2_^10^(LC-HRMS^1^) data set if the first Gaussian kernel width was reduced from 91 to 41, however peak splitting was observed for noisy chromatographic peaks. This issue may only affect isobaric ions. This can be seen in Figure 2D, where the original data set shows a single peak, but the *d*_2_^10^(LC-HRMS^1^) data set shows two peaks. In this instance, these correspond to two compounds with similar retention times but slightly different masses (Figure 2E,F).

**Figure 2:**
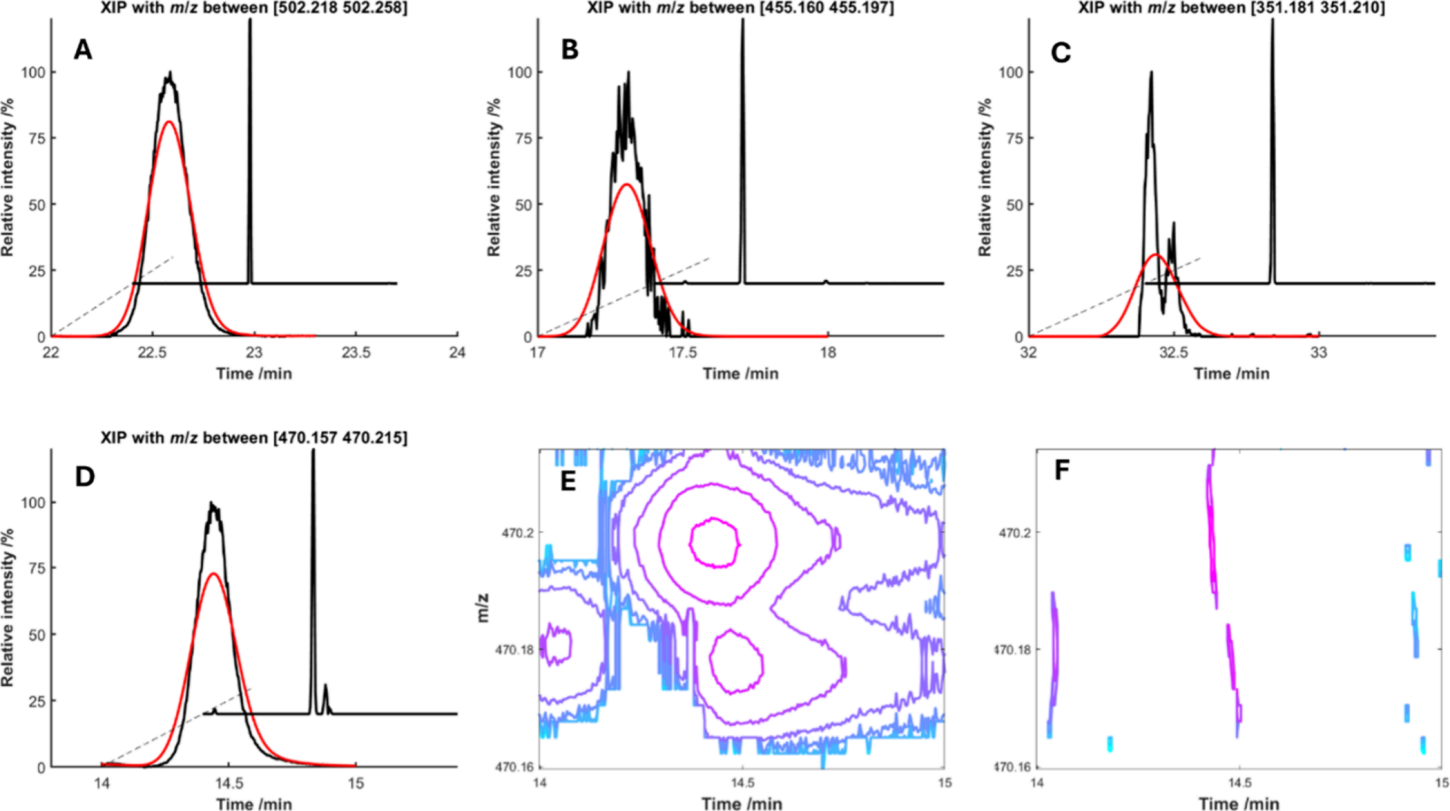
Superposition of the XIPs in the original data set on the first plan (before (black line) and after (red line) smoothing) and in the *d*_2_^10^(LC-HRMS^1^) data sets in the second plan, with *m*/*z* = 502.2384, 455.1755, 351.1951, and 470.1758 in panels (A–D), respectively. (E, F) Contour plots of the full data used in panel (D) in the original and the *d*_2_^10^(LC-HRMS^1^) data sets, respectively.

To comprehensively assess the derivative approach, targeted analyses were performed on derivative data sets generated with different cycle numbers: one cycle (*d*_2_^1^(LC-HRMS^1^)), five cycles (*d*_2_^5^(LC-HRMS^1^)) or ten cycles (*d*_2_^10^(LC-HRMS^1^)). XIPs were generated for each of the 836 compounds using the *m* ± Δ*m m*/*z* range. For each compound, a figure was created showing four quadrants: original data set XIC, *d*_2_^1^(LC-HRMS^1^) XIC, *d*_2_^5^(LC-HRMS^1^) XIC and *d*_2_^10^(LC-HRMS^1^) XIC. These figures are publicly available in a Zenodo repository https://zenodo.org/records/17055593); an example for compound 198 is provided in Figure S1.

Peak efficiency increased with the number of derivative cycles until it reached a maximum, which is dependent on the scan rate. Observations of peak shapes and peak efficiency calculations for each compound and data set are reported in Table S1. It should be noted that only 816 compounds are present in this table; 20 were excluded due to the absence of detectable peaks or signals below the limit of detection. The XIP centered around the *m*/*z* values of those 20 peaks can be found in the targeted analysis zip folder in the public repository (https://zenodo.org/records/17055593). Those are Figures #6, 11, 91, 224, 229, 231, 240, 265, 274, 277, 361, 403, 448, 449, 456, 459, 471, 556, 591, and 680. Most peaks in the original data set were of good quality, with 53 below the limit of quantification (LOQ). Fronting, tailing, and/or coelution were observed in 84 peaks, but did not hinder the effectiveness of the derivative approach. Only three cases of nonbaseline-separated peaks in the original data set resulted in single peaks in the derivative data set. Quantitatively, peak efficiency increased by an average factor of 2 between the original and *d*_2_^1^(LC-HRMS^1^) data sets, 11 between the original and *d*_2_^5^(LC-HRMS^1^), and 256 between the original and *d*_2_^10^(LC-HRMS^1^) data sets. This increase depends on the original scan number per peak, as derivative processing tends to equalize all peak widths to a similar number of scans per peak. For instance, compound 824, originally characterized by a very efficient peak, showed an initial efficiency loss (factor 0.6) after one derivative cycle but a final efficiency increase of 61-fold after 10 cycles.

### Untargeted Analysis with a Single File from the Bd1 Data Set

The primary benefit of derivative data sets is qualitative, enabling improved visualization of the number of independently separated compounds. Figure 3 illustrates this, showing the base peak profile (BPP) of SA1 in the original data set (Figure 3A) and in the *d*_2_^10^(LC-HRMS^1^) data set (Figure 3B). Pseudoenhanced peak efficiency, noise reduction, and baseline drift correction significantly improve visualization of individual analytes. For example, within the 8- to 10 min retention time window, five peaks are visible in the original data, whereas over 30 peaks are visible after derivation. To verify the method’s applicability, mass spectra at the apexes of five peaks within this time window were extracted from the *d*_2_^10^(LC-HRMS^1^) data set, and base peak ion extracted ion chromatograms (XICs) were retrieved from the original data set. The superimposed XICs (*m*/*z* values: 258.6010, 408.0206, 233.0922, 355.2244, and 371.1606) demonstrate the ability of this approach to separate complex chromatographic signals mathematically.

**Figure 3:**
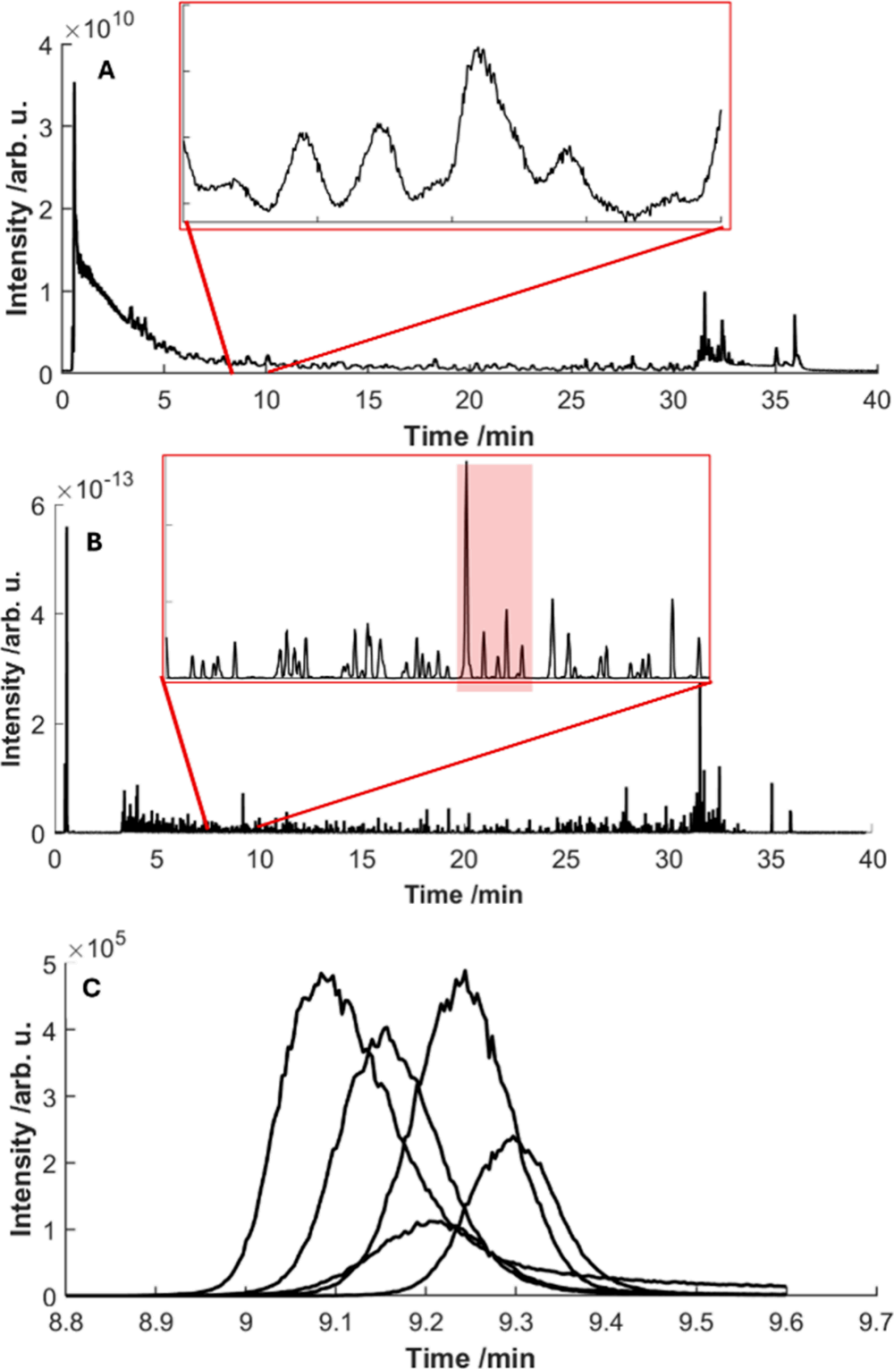
Base peak profiles (BPP) illustrating peak visualization improvement. (A) Original data set BPP of sample SA1. (B) *d*_2_^10^(LC-HRMS^1^) derivative data set BPP showing pseudoenhanced peak efficiency and baseline correction. (C) Superimposition of extracted ion chromatograms (XICs) from the original data set corresponding to base peak ions of the five peaks highlighted in panel XXXX­(B) (*m*/*z* values: 258.6010, 233.0922, 355.2244, and 371.1606). The boxes in panels (A, B) are a zoom of the BPPs between 8 and 10 min.

A peak picking algorithm was applied to the BPP of the *d*_2_^10^(LC-HRMS^1^) data set, detecting peak apexes and peak limits. MS spectra corresponding to each chromatographic peak were calculated by averaging all MS scans within peak boundaries. In total, 1,039 peaks were detected, spanning intensities over 5 orders of magnitude. Noise (*Nz*) was estimated using the 25th percentile.^27^ Of these peaks, 783 exceeded the noise threshold, 650 exceeded the limit of detection (LOD), and 511 exceeded the limit of quantification (LOQ). By comparison, the original data set yielded only 169 peaks, of which 36 exceeded the LOQ. Notably, many small peaks in the *d*_2_^10^(LC-HRMS^1^) data set shared base peak ions in their MS spectra. These arise from the cyclic derivation of background ions, as shown in Figure S2. While background ions with perfect linear profiles disappear after derivation, those with varying intensity profiles may produce ghost peaks. A routine was developed to identify and remove problematic background ions by nullifying ions within *m* ± Δ*m m*/*z* windows in the original data set. Although 37 ions were detected, only 35 were removed to preserve data integrity. After removal, 1,059 peaks were detected, with 803 above noise, 656 above LOD, and 553 above LOQ. After removing the background ions, the noise was reduced by approximately a factor of 2. The final BPP and peak-picking results are available in the public Zenodo repository as TIFF and FIG files (https://zenodo.org/records/17055593).

The untargeted peak picking approach automatically detects 650 peaks above the LOD, calculates their limits, and obtains average MS spectra. A second peak-picking procedure calculates the *m*/*z* positions and intensities of ions within each spectrum. Peaks are tentatively assigned to one of the 816 known compounds based on isotopologue patterns obtained via ChemCalc (see Table S1). Two levels of identification were employed: Level 1 if two ions in a peak matched the [M] + H^+^ and [M + 1] + H^+^ isotopologues within 10 and 15 ppm absolute mass error, respectively; Level 2 if the relative intensity error between these ions and the theoretical ChemCalc ratio was less than 40%. Results summarized in Table S1 show detection of 790 compounds at Level 1 and 748 at Level 2. The original relative mass errors of 10 ppm for [M] + H^+^ and 15 ppm for [M + 1] + H^+^, used to match experimental base peaks to theoretical values from ChemCalc, are larger than typical Orbitrap tolerances (∼5 ppm). However, as shown in Table S1, applying a stricter 5 ppm limit to both isotopologues would reduce Level 1 identifications by 6 compounds and Level 2 identifications by 19 compounds. These findings demonstrate that the derivative data sets improve peak efficiency and reduce background ion impact while preserving isotopologues, adducts, and fragments of single compounds within single peaks. Despite enhanced peak efficiency, significant comigration remains; for example, Peak 4 contains 29 compounds and Peak 34 contains 10 compounds. Detailed results and figures are available in the Zenodo repository and Supporting Information (e.g., Figure S3). Each detected peak figure comprises four panels: (1) peak and limits in BPP, (2) average MS spectrum from *d*_2_^10^(LC-HRMS^1^), (3) overlay of original and derivative XIPs for the most intense ion, and (4) list of most intense ions with *m*/*z* and relative intensities of their first isotopologues. The high mathematical separation capacity of this transformation is illustrated in Figure 4, where ions at *m*/*z* 188.5850, 189.0867, 376.1629, and 377.1659 correspond to [M] + 2H^+^, [M + 1] + 2H^+^, [M] + H^+^, and [M + 1] + H^+,^ where M is C_18_H_25_N_5_S_2_. Insets compare experimental and theoretical isotopograms for the doubly and singly protonated species.

**Figure 4:**
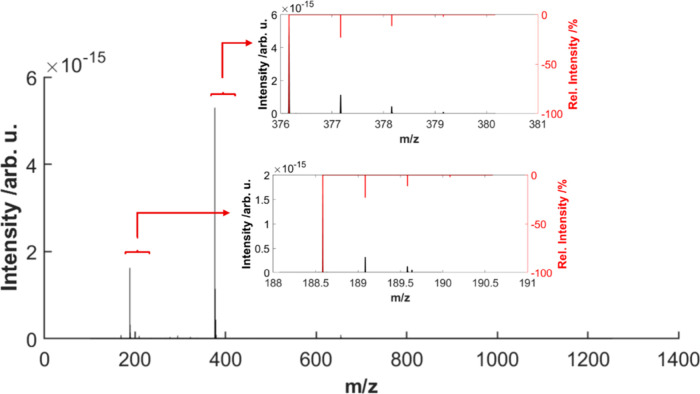
Mass spectra obtained by averaging MS scans of peak 191 in the *d*_2_^10^(LC-HRMS^1^) data set (retention time 9.427–9.480 min). Insets figures show zoomed experimental and reversed theoretical isotopograms for ions C_18_H_27_N_5_S_2_^2+^ (doubly protonated) and C_18_H_26_N_5_S_2_^+^ (singly protonated).

To assess whether the cyclic derivative steps modified peak positions, Finnee results were compared with feature figures of merit obtained using XCMS as a benchmark approach.^10,11^ The XCMS peak table was generated using the default parameters for the uHPLC/Orbitrap setting (parameter #137), yielding 12,679 features. Features were matched when times and *m*/*z* at peaks apexes differed by less than 0.5 min and 5 ppm, respectively. Of the 748 compounds listed in Table S1, 92.6% matched XCMS features, with a mean apex time difference of 0.00 ± 0.05 min (SD, *n* = 693) and a mean relative mass difference of 0.0 ± 0.7 ppm (SD, *n* = 693), showing that the successive derivative steps did not significantly change the apex position in either dimension.

### Analysis with All Files from the Bd1 Data Set

The pseudoenhancement approach was applied to all 10 files of the Bd1 data set (replicates SA*x* and SB*x*, *x* = 1.5). Overlapping TIPs of the *d*_2_^10^(LC-HRMS^1^) data sets from SA replicates (Figures S4–S8) show consistent profiles, demonstrating method reliability in enhancing peak efficiency, reducing background ions, and decreasing noise. Using the replicates and 748 test compounds detected at Level 2 in untargeted analysis, the repeatability of peak area measurements was evaluated in the original data set and derivative data set (*d*_2_^1^(LC-HRMS^1^), *d*_2_^5^(LC-HRMS^1^) and *d*_2_^10^(LC-HRMS^1^)). The precision was assessed by relative standard deviation (RSD) of concentration ratios between SA and SB replicates compared to theoretical ratios from Li et al.^13^ Results (Table 1) indicate slightly worsened repeatability in derivative data sets, likely due to reduced data points per peak and cumulative mathematical processing. However, the impact is small: RSD was 5.7% for SA in original data versus 6.6% in *d*_2_^10^(LC-HRMS^1^). Interestingly, the relative standard error (RSE) of concentration ratios improved with derivative data sets, possibly due to more consistent peak limit detection.

**Table 1:** Accuracy and Precision of Peak Area Measurements in the Original and Derivative Data Sets^a^

Data set	RSD (SA*x*)	RSD (SB*x*)	RSE (SB*x*/SA*x*)
original	5.7%	5.1%	3.5%
*d*_2_^1^(LC-HRMS^1^)	6.1%	5.6%	2.5%
*d*_2_^5^(LC-HRMS^1^)	6.5%	6.0%	2.4%
*d*_2_^10^(LC-HRMS^1^)	6.6%	6.0%	2.4%

a Relative standard deviation (RSD) is given for replicates SA*x* and SB*x*, along with relative standard error (RSE) for concentration ratios SB*x*/SA*x*. The data sets correspond to the original data and derivative data sets with 1, 5, and 10 derivative cycles.

Those results demonstrate the potential of derivative data sets to improve visualization and to reveal isotopologue and adduct patterns for many compounds, even when chromatographic separation is poor. However, optimizing the kernel size for each cycle would be time-consuming. Preliminary results suggest that good performance can be obtained by starting with a kernel size approximately equal to the average number of scans per peak in the chromatographic dimension, followed by a 20–30% decrease in kernel size for each cycle. To avoid shifting the peak apex, the kernel size should be an odd integer. Figure S9 shows the evolution of the TICs for the SA1 files for different numbers of cycles, always starting with a kernel size of 91. As can be seen, each additional cycle slightly improves peak efficiency.

### Untargeted Analysis with the EBC File

The derivative approach was applied to the EBC sample representing a real complex biological matrix. Optimization of pseudoenhanced efficiency was achieved after eight cycles with Gaussian kernel widths: 61, 41, 31, 21, 11, 9, 7, and 5 scans. A total of 633 peaks were automatically detected in the BPP of the *d*_2_^8^(LC-HRMS^1^) data set, among which 555 had signal-to-noise ratios (*S*/*N*) greater than 3, and 116 had *S*/*N* > 10. Automatic background ion removal detected 19 ions, removing 13 to prevent loss of meaningful data (*m*/*z* = 229.1428, 201.1125, 83.0603, 99.5309, 129.0545, 158.9610, 90.5258, 111.0435, 246.1681, 88.0232, 125.9862, 143.9970, and 102.0336). The MS spectra in the EBC derivative data set were noisier than those in Bd1, presumably due to the presence of many components at low or very low concentrations. Nonetheless, 57 peaks in the BPP have MS spectra that contain at least two isotopologues of the same ion, and 10 peaks in the BPP have MS spectra with at least two adducts of the same parent compound, each with their respective isotopologues (see the EBC Untargeted Analysis zip folder in https://zenodo.org/records/17055593). Peaks #75 and #80 were notable for displaying five visible adducts differing by *m*/*z* 28.0315, indicating related components differing only by C_2_H_4_. While compound identification was not the aim of this study, these results demonstrate the remarkable mathematical separation capability of the proposed approach.

## Practical Limitation and Potential Artifacts

The derivative-based workflow relies on repeated smoothing and second-derivative operations, which are intrinsically nonlinear and can introduce artifacts if parameters are not carefully optimized. When multiple cycles are applied, local fluctuations that are not chromatographic peaks may be sharpened into narrow features, particularly for background ions. Users should exercise caution when encountering small, isolated peaks and verify them in the original data. To mitigate this, the implementation includes the detection and removal of background ions that generate families of “ghost” peaks.

Also, because the transformation combines smoothing, derivation, inversion, and truncation of negative values, absolute areas are not preserved and should not be interpreted as a linear filter. Derivative data sets are therefore best used as a complement to the original data, providing a qualitative aid for feature detection and feature grouping. More accurate quantitative results are obtained by using the original, untransformed data with a targeted approach. The method also has intrinsic constraints regarding chromatographic resolution. It cannot recover separate peaks when two perfectly isobaric analytes are unresolved in the original data. In rare cases, a nonbaseline-separated doublet may appear as a single, sharpened feature, depending on the chosen Gaussian kernel widths. Results should always be cross-checked against extracted profiles from the original data set.

Finally, the workflow imposes practical requirements on data and computing resources. It is designed for profile-mode acquisitions with high sampling rates per peak. Data sets with few scans per chromatographic peak will be less suitable for multiple derivative cycles. Moreover, the cyclic processing of large matrices is computationally intensive. Users should balance the desired degree of pseudoenhancement against available computing time and the characteristics of the mass analyzer.

## Conclusions

This study presents a robust algorithmic workflow enabling pseudoenhancement of peak efficiency in LC-HRMS^1^ data sets by means of multiple derivative-based transformations and Gaussian smoothing. This hardware-independent approach significantly improves peak resolution, visualization, and feature detection, while preserving crucial isotopologue and adduct patterns needed for compound annotation. Some minor trade-offs arise (notably, a modest decrease in repeatability due to data point reduction), but the overall impact is positive. The proposed method can be integrated into future analytical workflows to address persistent data processing challenges in untargeted high-resolution mass spectrometry. However, the approach only works with profile scans, which involve large data sets, and the cyclic repetition of the Gaussian smoothing filter and the second derivative makes it computationally intensive. Moreover, the best results are obtained with high sampling rates, making computing time the most limiting factor. For example, on a personal computer, with Bd1 files that, on average, contain 11,900 MS scans each with 550,000 data points, the 10 cycles were processed in 125 min. By comparison, the EBC file, which contains 5,929 MS scans, each with 722,000 data points, was processed (8 cycles) in 42 min.

It should be noted that this approach is a pseudoenhancement rather than a true enhancement. When two isobaric peaks are heavily overlapped and cannot be seen as distinct entities (with a resolution of 0.5 or lower), no improvement in resolution can be expected. For peaks that are not baseline-separated but remain distinguishable, the gain depends not only on their resolution but also on the width of the Gaussian smoothing filter. A key limitation is that the same width is applied for all Gaussian smoothing operations. Although this width was optimized for the average peak, it may oversmoothed very narrow peaks, which commonly occur at the beginning of the separation. However, as implemented in this study, such effects can be readily visualized and accounted for. This issue is only critical for perfectly isobaric peaks. As demonstrated in this work, even a small *m*/*z* difference is sufficient to mathematically separate comigrating peaks

While the pseudoenhancement approach is primarily a visualization and mathematical separation method that helps obtain clean isotopologue patterns for many compounds present in the original data set, it is also orthogonal to existing HPLC-HRMS computational tools. Used in combination with these tools, it may improve the detection of false positives.

## Supplementary Material





## Data Availability

The new release of Finnee2024 with the functions used in this manuscript can be downloaded at https://github.com/glerny/Finnee2024/releases/tag/v0.10.01. Figures supporting the findings of this study, including all targeted analysis, background ion analyses, untargeted analysis are available on Zenodo (https://zenodo.org/records/17055593). The data are provided as zip folders containing TIFF and MATLAB FIG files.

## Footnotes

1 To be consistent with all separation techniques (chromatography, electrochromatography, and electrophoresis), the term profile is used instead of chromatography, thus TIP is used rather than TIC, BPP rather than BPC, and XIP rather than XIC.

## Notes

Additional information and figures related to this work, including a figure generated by Finnee for each compound in thetargeted analysis, an ion detected as a background ion, a figure generated by Finnee for each peak detected in theuntargeted analysis, etc. (PDF)
Table S1: Information on targeted compounds in the Bd1 data set and results in the targeted approach (XLSX)
