# Supplementary material for: Pseudoenhanced Peak Efficiency in Liquid Chromatography–High Resolution Mass Spectrometry Using Multiple Second Derivatives
Source: J Am Soc Mass Spectrom. 2026 Jun 5;37(7):1519–26. doi: 10.1021/jasms.5c00315 (PMC13329988; doi:10.1021/jasms.5c00315)
Supplement: Supplementary file 1 [file js5c00315_si_001.pdf]

Supporting information

Pseudo-enhanced peak efficiency in LC-HRMS using multiple second-derivatives.

Author names Guillaume Laurent Erny*[a,b]

[a] Associate Laboratory i4HB - Institute for Health and Bioeconomy, University Institute of Health Sciences - CESPU, 4585-116 Gandra, Portugal.

[b] UCIBIO - Applied Molecular Biosciences Unit, Translational Toxicology Research Laboratory, University Institute of Health Sciences (1H-TOXRUN, IUCS-CESPU), 4585-116 Gandra, Portugal.

Email: guillaume.erny@iucs.cespu.pt

Table of Contents

S1. Short tutorial	S3
S2. Pseudocode for the Cyclic Derivative	S5
Figure S1. Example of a figure generated by Finnee for each compound in the targeted analysis. Extracted ion profiles (XIPs) of the original LC-HRMS¹ dataset and derivative datasets $d_2^1, d_2^5,$ and d_2^{10} are shown. Peak limits are indicated. Gaussian filter widths vary with each derivative cycle, resulting in progressively smoother and enhanced peaks.	S6
Figure S2. Example of an ion detected as a background ion. The top panel shows the extracted ion profile (XIP) in the original dataset, and the bottom panel shows the corresponding profile in the d_2^{10} $(\mathrm{LC}-\mathrm{HRMS}^1)$ dataset.	S6
Figure S3. Example figure generated by Finnee for each peak detected in the untargeted analysis. The base peak profile (BPP) is displayed with peak limits, averaged mass spectra within peak boundaries, and annotations of principal adducts and isotopologues.	S7
Figure S4. Comparison of total ion profiles (TIPs) between the original dataset (left) and d_2^{10} $(\mathrm{LC}-\mathrm{HRMS}^1)$ derivative dataset (right) for SA replicate files (SA1 to SA5). Retention time window: 0 to 4 minutes. Each peak in the right window corresponds to one or more chemicals. The enhanced XIP and classical XIP obtained from each base peak ion can be seen on the Zenodo repository (https://zenodo.org/records/17055593), in the Untargeted analysis zip folder (Figures\TIFF) (Figure #1 to Figure #52 in order of elution).	S7
Figure S5. Comparison of total ion profiles (TIPs) between the original dataset (left) and d_2^{10} $(\mathrm{LC}-\mathrm{HRMS}^1)$ derivative dataset (right) for SA replicate files (SA1 to SA5). Retention time window: 4 and 8 min. Each peak in the right window corresponds to one or more compounds. The enhanced XIP and classical XIP obtained from each base peak ion can be seen on the Zenodo repository (https://zenodo.org/records/17055593), in the Untargeted analysis zip folder (Figures\TIFF) (Figure #53 to Figure #161 in order of elution).	S8
Figure S6. Comparison of total ion profiles (TIPs) between the original dataset (left) and d_2^{10} $(\mathrm{LC}-\mathrm{HRMS}^1)$ derivative dataset (right) for SA replicate files (SA1 to SA5). Retention time window: 8 and 12 min. Each peak in the right window corresponds to one or more compounds. The enhanced XIP and classical XIP obtained from each base peak ion can be seen on the Zenodo repository (https://zenodo.org/records/17055593), in the Untargeted analysis zip folder (Figures\TIFF) (Figure #161 to Figure #241 in order of elution).	S9
Figure S7 Comparison of total ion profiles (TIPs) between the original dataset (left) and d_2^{10} $(\mathrm{LC}-\mathrm{HRMS}^1)$ derivative dataset (right) for SA replicate files (SA1 to SA5). Retention time window: 12 and 16 min. Each peak in the right window corresponds to one or more compounds. The enhanced XIP and classical XIP obtained from each base peak ion can be seen on the Zenodo repository (https://zenodo.org/records/17055593), in the Untargeted analysis zip folder (Figures\TIFF) (Figure #242 to Figure #318 in order of elution).	S1<br>0
Figure S8. Comparison of total ion profiles (TIPs) between the original dataset (left) and d_2^{10} $(\mathrm{LC}-\mathrm{HRMS}^1)$ derivative dataset (right) for SA replicate files (SA1 to SA5). Retention time window: 16 and 20 min. . Each peak in the right window corresponds to one or more compounds. The enhanced XIP and classical XIP obtained from each base peak ion can be seen on the Zenodo repository (https://zenodo.org/records/17055593), in the Untargeted analysis zip folder (Figures\TIFF) (Figure #319 to Figure #373 in order of elution).	S1<br>1
Figure S9. Comparison of total ion profiles (TIPs) for SA1 for different number of derivative cycles: (a) original data, (b) $d_2^1(\mathrm{LC}-\mathrm{HRMS}^1)$, (c) $d_2^2(\mathrm{LC}-\mathrm{HRMS}^1)$, (d) $d_2^3(\mathrm{LC}-\mathrm{HRMS}^1)$, (e) d_2^4 $(\mathrm{LC}-\mathrm{HRMS}^1)$, (f) $d_2^5(\mathrm{LC}-\mathrm{HRMS}^1)$, (g) $d_2^6(\mathrm{LC}-\mathrm{HRMS}^1)$, (h) $d_2^7(\mathrm{LC}-\mathrm{HRMS}^1)$, (i) $d_2^8(\mathrm{LC}-\mathrm{HRMS}^1)$, (j) $d_2^9(\mathrm{LC}-\mathrm{HRMS}^1)$, (k) $d_2^{10}(\mathrm{LC}-\mathrm{HRMS}^1)$. Retention time window: 10 to 15 min.	S1<br>2

S1. Short tutorial

Step 1: Creating the Finnee Object

```
>> myFinnee = Finnee();
```

- Finnee is the constructor for creating a Finnee object containing all MS scans and metadata information.
- After calling this function, you will be asked to select an mzML file (this is the only supported format). Only MS1 and profile scans are recorded.
- All scans from a single separation are stored in one dataset.
- Data transformations are recorded as new, successive datasets.
- Naming your object myFinnee is recommended for consistency.

Step 2: 2D Interpolation of MS Scans

```
>>  XY = myFinnee.getProfile(1, 'Total_Ions_Profile').Data;
```

- This retrieves the Total Ion Profile (TIP) for the first dataset (which consists of the raw MS scans from the mzML file).
- XY is a two-column matrix:
    - Column 1: Time at which each scan was acquired.
    - Column 2: Total ion intensity per scan (sum of all ion intensities in the spectrum).

```
>> plot(XY(:, 1), XY(:, 2)); % Plot the TIP
>> plot(diff(XY(:, 1)));     % Visualize the scan-to-scan time intervals
```

To create a uniform time axis with constant intervals:

```
>> AxisTm = min(XY(:, 1)):mean(diff(XY(:, 1))):max(XY(:, 1));
>> myAxis.TimeAxis = AxisTm;
```

To Interpolate all MS scans onto this new time axis and a master m/z axis:

```
>> myFinnee = myFinnee.Interpolate2D(1, myAxis);
```

- The master m/z axis is estimated using the most intense scan by default, or you can specify it: myAxis.mzAxis = Axismz;
- Interpolated MS scans are saved as Dataset 2.

Step 3: Cyclic Derivative Transformation

```
>> myFinnee = myFinnee.mkDerivatives(2, 'cycles', [61, 41, 31, 21]);
```

- Applies a cyclic derivative with respect to scan number (time) to all MS scans in Dataset 2.
- Each kernel size in the vector defines the Gaussian smoothing filter width for one cycle. The number of cycles is the length of this vector.

Optional: Remove background ions

```
>> Ions  = [229.1428, 201.1125, 83.0603, 99.5309, ...
     129.0545, 158.9610, 90.5258, 111.0435, 246.1681, 88.0232, ...
     125.9862, 143.9970, 102.0336];
>> myFinnee = myFinnee.mkDerivatives(2, 'cycles', [61, 41, 31, 21, 11, 9, 7, 5], ...
  'Dmz', 50000, 'IonsOut', Ions);
```

- This example uses 8 cycles and removes all intensities within the m/z range $(m/z)_i \pm 4 \times (m/z)_i/Dmz$.

Data Visualization Examples

1. Total Ion Profile (TIP) and Base Peak Profile (BPP):

```
>> prf = myFinnee.getProfile(dts, 'Total_Ions_Profile');
>> prf = myFinnee.getProfile(dts, 'Base_Peak_Profile');
>> prf.plot;   % Plot the profile
>> prf.Data;   % Access underlying data
```

   - dts is the dataset index: 1 = raw, 2 = interpolated, 3 = derivatives.

2. Averaged MS scans:

```
>> scn = myFinnee.mkSpectrum(dts, [t_start t_end]);
```

   - Returns the averaged MS for scans acquired between t_start and t_end (not valid for Dataset 1).

3. Extracted Ion Profile (XIP):

```
>> XIP = myFinnee.mkProfile(dts, [mz_start mz_end]);
```

   - Extracts the ion profile from mz_start to mz_end.

S2. Pseudocode for the Cyclic Derivative

Inputs:

- myFinnee: Finnee Object

- dts: Dataset index (must reference interpolated scans on a common m/z and time axis)
- KSz: Vector (number of cycles; kernel size for each Gaussian smoothing filter)
- Optional:
    - Dmz: Theoretical resolving power
    - Ions2rem: List of background ion m/z values

Algorithm:

1. Load the first block of MS spectra from the dataset into myData matrix.
2. If Ions2rem is not empty, filter out ions in Ions2rem (within ± interval set by Dmz).
3. For ii, each kernel size in KSz:
    - Smooth each row with a Gaussian filter of the given kernel size:
        - myData = smoothdata2(myData, 'gaussian', {1, KSz(ii)})
    - Compute the second derivative along scan number, multiply by -1:
        - [~, g] = sgolay(2, 3);
        - myData = -filter2(g(:, 3)', myData, 'same');
    - Set negative values to zero.
4. Save each column of myData as a new MS scan in a new dataset.
5. Repeat for all scan blocks.

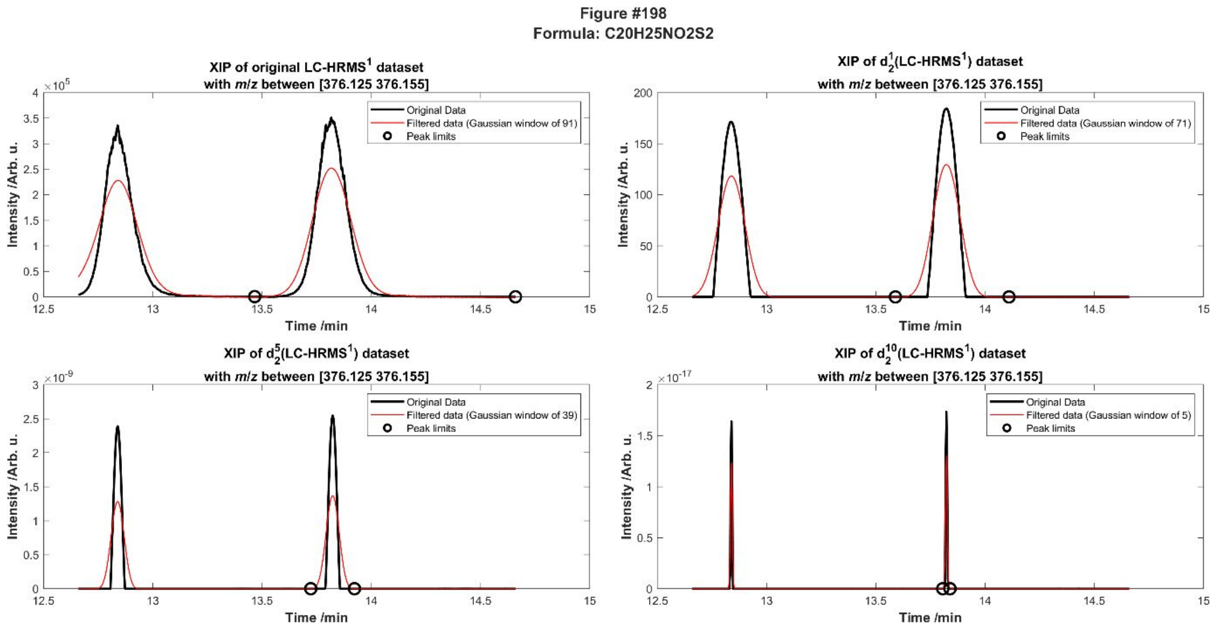

Figure S1. Example of a figure generated by Finnee for each compound in the targeted analysis. Extracted ion profiles (XIPs) of the original LC-HRMS[1] dataset and derivative datasets d_2^1, d_2^5, and d_2^{10} are shown. Peak limits are indicated. Gaussian filter widths applied vary with each derivative cycle, demonstrating progressive peak smoothing and enhancement.

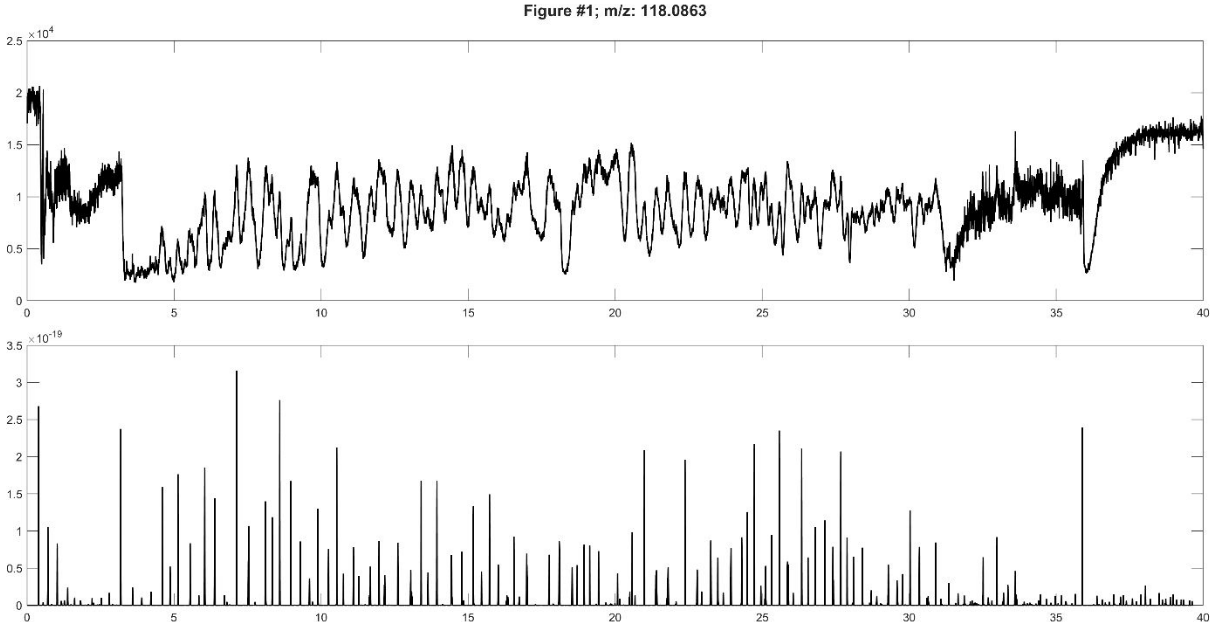

Figure S2. Example of an ion detected as a background ion. The top panel shows the extracted ion profile (XIP) in the original dataset, and the bottom panel shows the corresponding profile in the $d_2^{10}(\mathrm{LC-HRMS^1})$ dataset.

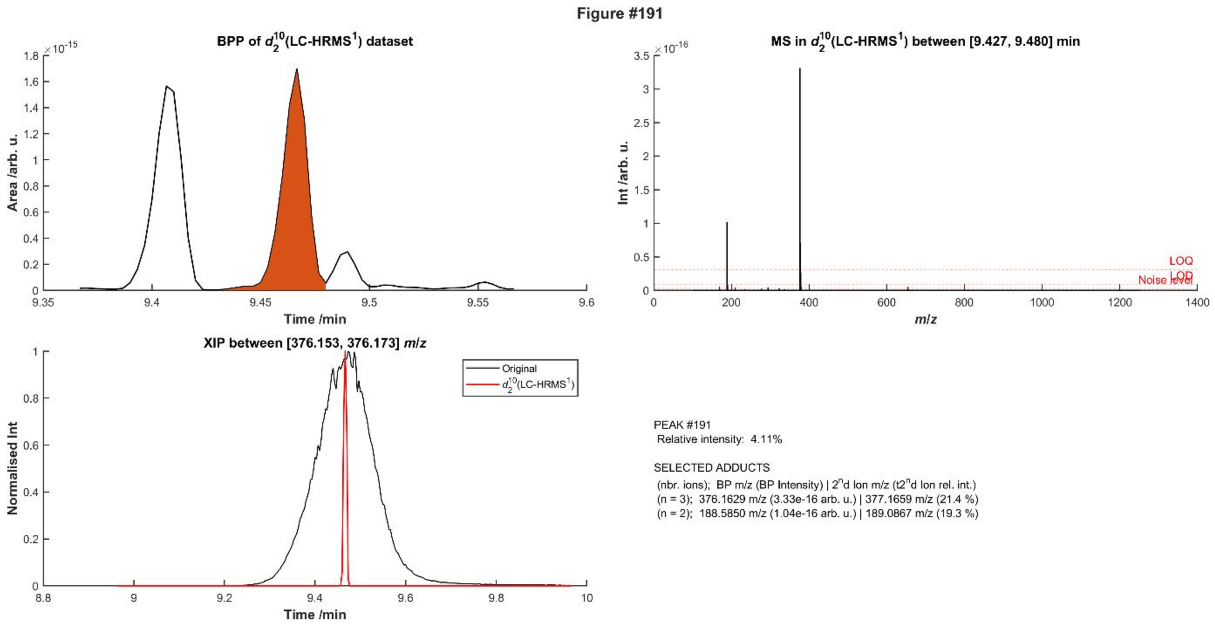

Figure S3. Example figure generated by Finnee for each peak detected in the untargeted analysis. The base peak profile (BPP) is displayed with peak limits, averaged mass spectra within peak boundaries, and annotations of principal adducts and isotopologues.

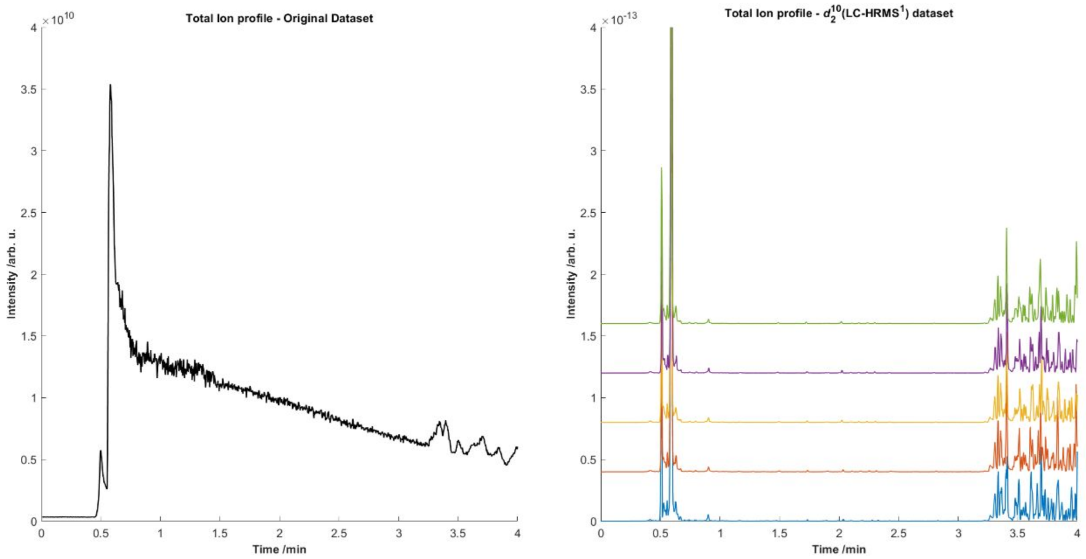

Figure S4. Comparison of total ion profiles (TIPs) between the original dataset (left) and $d_2^{10}(\mathrm{LC}-\mathrm{HRMS}^1)$ derivative dataset (right) for SA replicate files (SA1 to SA5). Retention time window: 0 to 4 minutes. Each peak in the right window corresponds to one or more chemicals. The enhanced XIP and classical XIP obtained from each base peak ion can be seen on the Zenodo repository (https://zenodo.org/records/17055593), in the Untargeted analysis zip folder (Figures\TIFF) (Figure #1 to Figure #52 in order of elution).

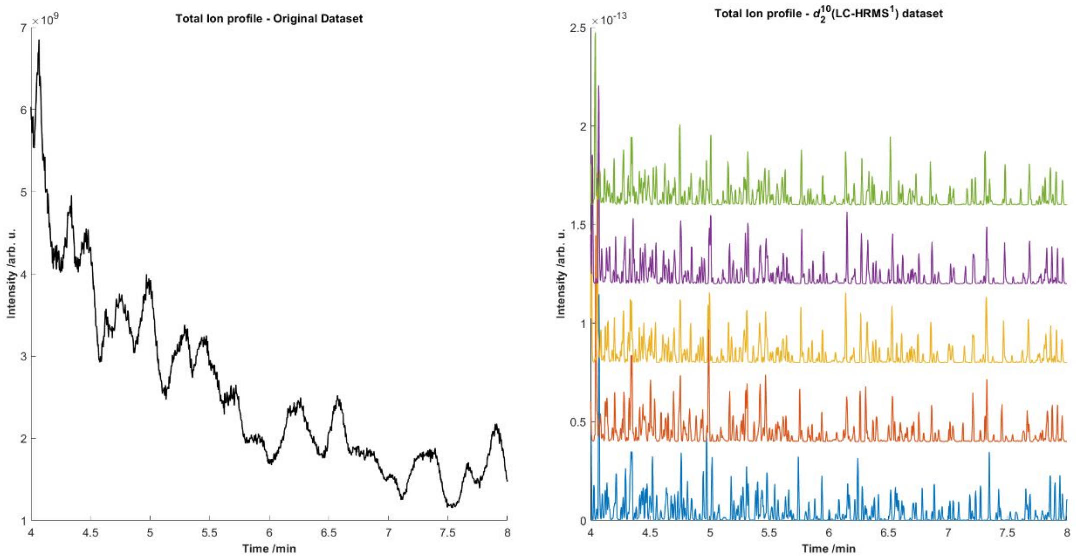

Figure S5. Comparison of total ion profiles (TIPs) between the original dataset (left) and $d_2^{10}(\mathrm{LC-HRMS^1})$ derivative dataset (right) for SA replicate files (SA1 to SA5). Retention time window: 4 and 8 min. Each peak in the right window corresponds to one or more chemicals. The enhanced XIP and classical XIP obtained from each base peak ion can be seen on the Zenodo repository (https://zenodo.org/records/17055593), in the Untargeted analysis zip folder (Figures\TIFF) (Figure #53 to Figure #161 in order of elution).

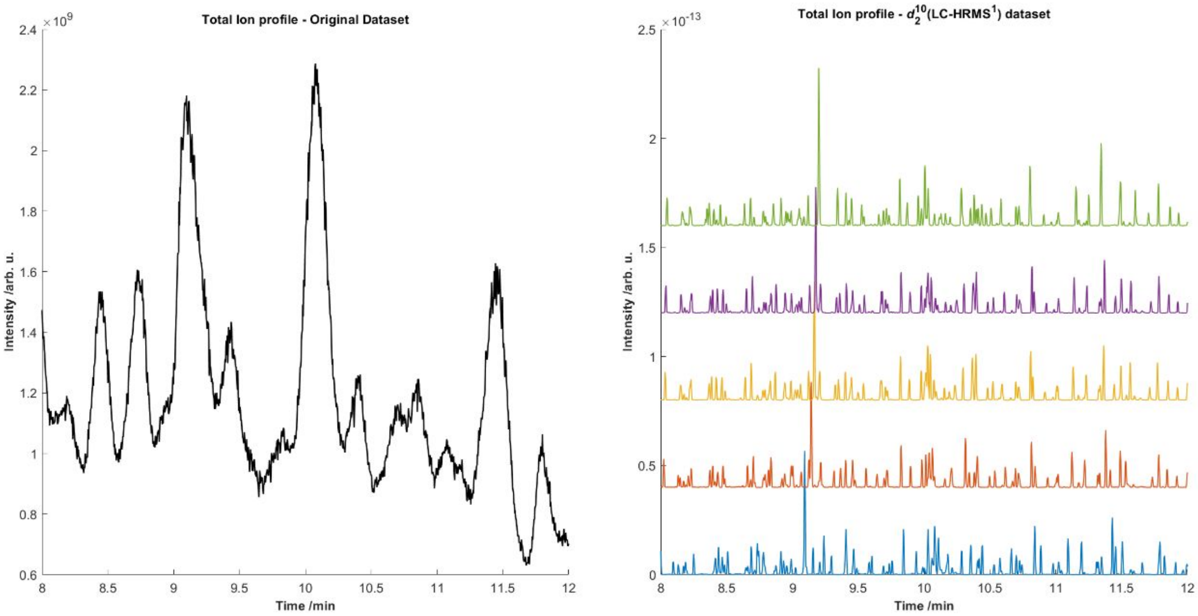

Figure S6. Comparison of total ion profiles (TIPs) between the original dataset (left) and $d_2^{10}(\mathrm{LC}-\mathrm{HRMS}^1)$ derivative dataset (right) for SA replicate files (SA1 to SA5). Retention time window: 8 and 12 min. Each peak in the right window corresponds to one or more chemicals. The enhanced XIP and classical XIP obtained from each base peak ion can be seen on the Zenodo repository (https://zenodo.org/records/17055593), in the Untargeted analysis zip folder (Figures\TIFF) (Figure #161 to Figure #241 in order of elution).

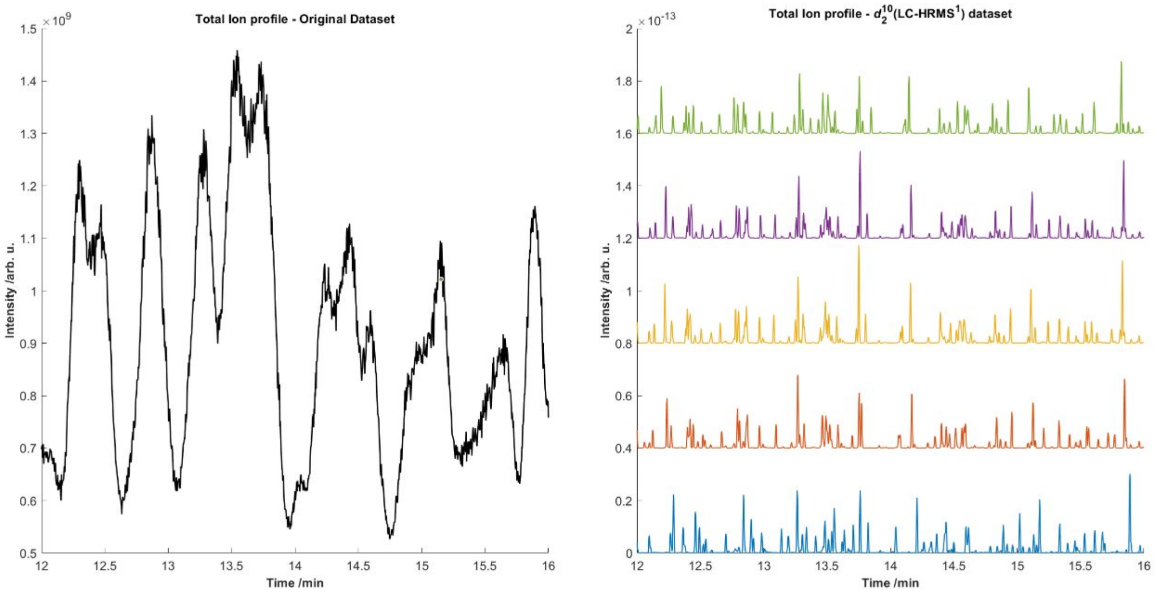

Figure S7. Comparison of total ion profiles (TIPs) between the original dataset (left) and $d_2^{10}(\mathrm{LC}-\mathrm{HRMS}^1)$ derivative dataset (right) for SA replicate files (SA1 to SA5). Retention time window: 12 and 16 min. Each peak in the right window corresponds to one or more chemicals. The enhanced XIP and classical XIP obtained from each base peak ion can be seen on the Zenodo repository (https://zenodo.org/records/17055593), in the Untargeted analysis zip folder (Figures\TIFF) (Figure #242 to Figure #318 in order of elution).

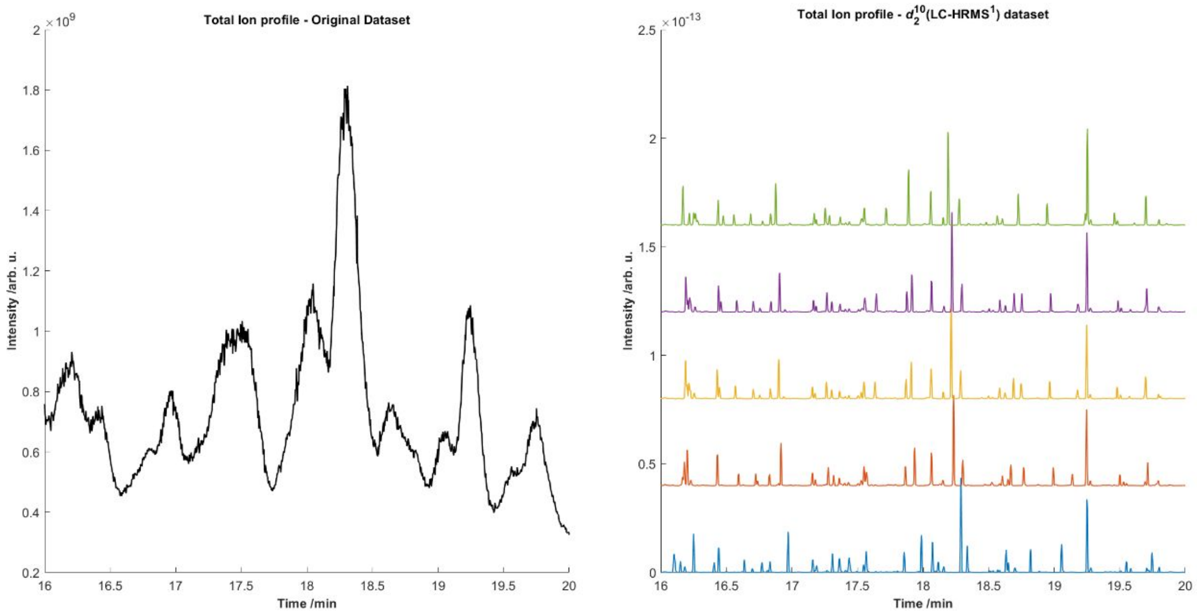

Figure S8. Comparison of total ion profiles (TIPs) between the original dataset (left) and $d_2^{10}(\mathrm{LC}-\mathrm{HRMS}^1)$ derivative dataset (right) for SA replicate files (SA1 to SA5). Retention time window: 16 and 20 min. . Each peak in the right window corresponds to one or more chemicals. The enhanced XIP and classical XIP obtained from each base peak ion can be seen on the Zenodo repository (https://zenodo.org/records/17055593), in the Untargeted analysis zip folder (Figures\TIFF) (Figure #319 to Figure #373 in order of elution).

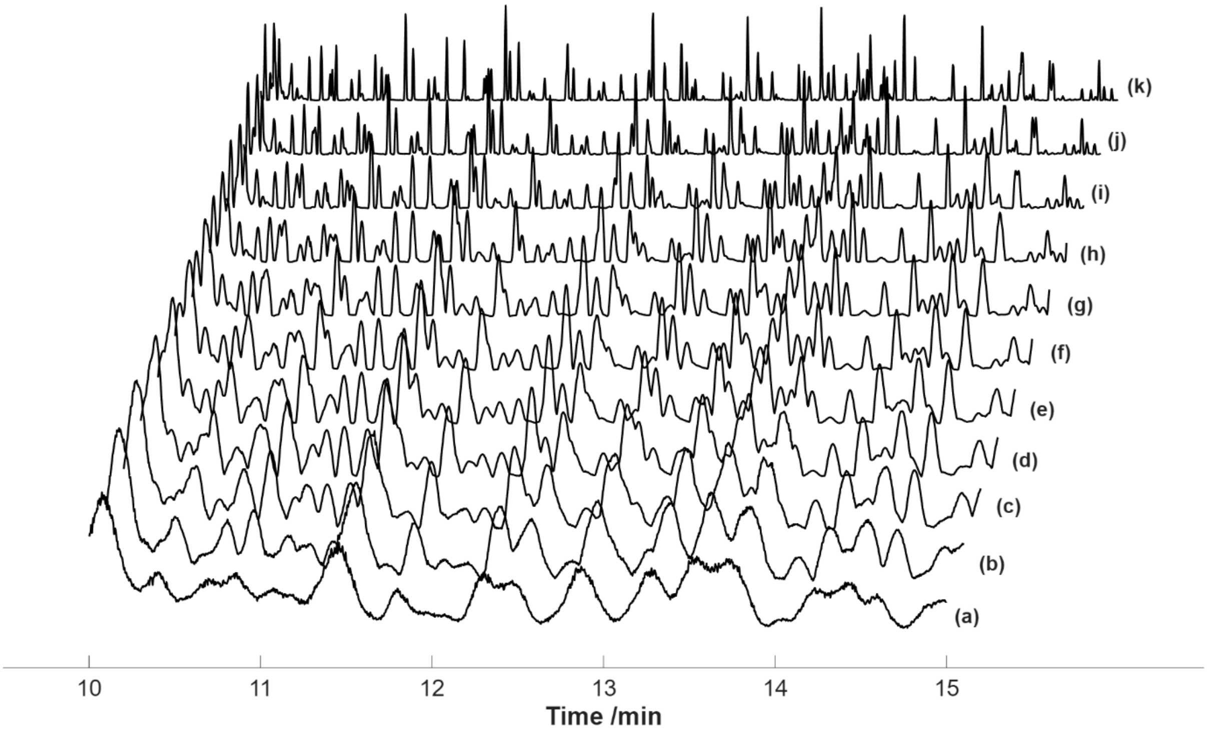

Figure S9. Comparison of total ion profiles (TIPs) for SA1 for different number of derivative cycles: (a) original data, (b) $d_2^1(\mathrm{LC-HRMS^1})$, (c) $d_2^2(\mathrm{LC-HRMS^1})$, (d) d_2^3 $(\mathrm{LC-HRMS^1})$, (e) $d_2^4(\mathrm{LC-HRMS^1})$, (f) $d_2^5(\mathrm{LC-HRMS^1})$, (g) $d_2^6(\mathrm{LC-HRMS^1})$, (h) $d_2^7(\mathrm{LC-HRMS^1})$, (i) $d_2^8(\mathrm{LC-HRMS^1})$, (j) $d_2^9(\mathrm{LC-HRMS^1})$, (k) $d_2^{10}(\mathrm{LC-HRMS^1})$. Retention time window: 10 to 15 min.
